# Supplementary material for: Construction and clinical application of a risk model based on N6-methyladenosine regulators for colorectal cancer
Source: PeerJ. 2024 Dec 20;12:e18719. doi: 10.7717/peerj.18719 (PMC11665428; doi:10.7717/peerj.18719)
Supplement: Supplemental Information 1 — (A) Differences in clinical characteristics of three subtypes in AC-ICAM cohort. (*p<0.05, **p<0.01, ***p<0.001) [file peerj-12-18719-s001.pdf]

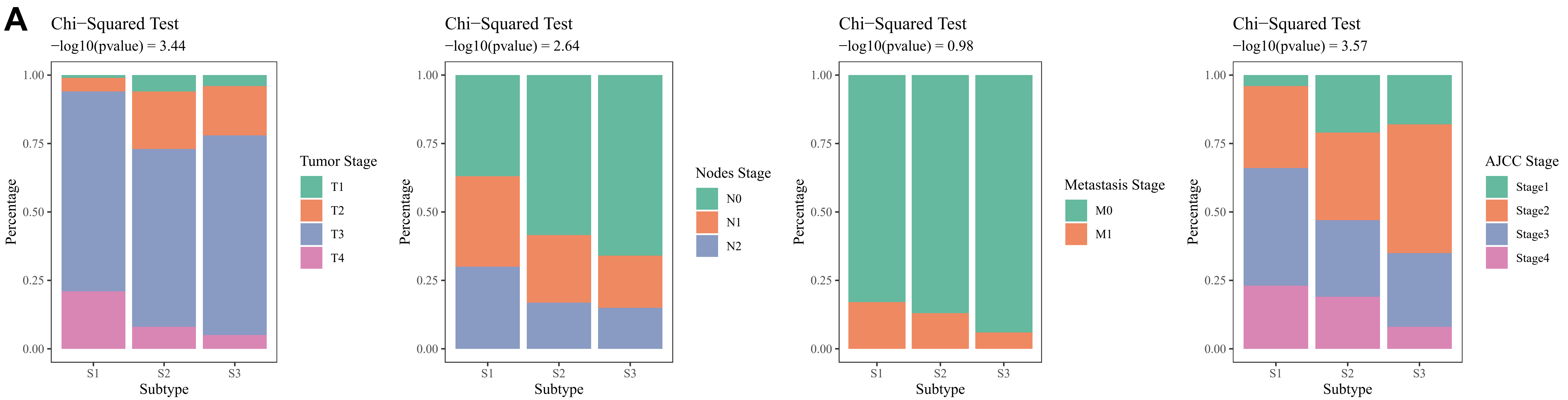
A
Chi−Squared Test
−log10(pvalue) = 3.44
Tumor Stage
T1
T2
T3
T4
Chi−Squared Test
−log10(pvalue) = 2.64
Nodes Stage
N0
N1
N2
Chi−Squared Test
−log10(pvalue) = 0.98
Metastasis Stage
M0
M1
Chi−Squared Test
−log10(pvalue) = 3.57
AJCC Stage
Stage1
Stage2
Stage3
Stage4
Percentage
1.00
0.75
0.50
0.25
0.00
S1
S2
S3
Subtype
